# Human islet function following 20 years of cryogenic biobanking

**DOI:** 10.1007/s00125-015-3598-4

**Published:** 2015-05-01

**Authors:** Jocelyn E. Manning Fox, James Lyon, Xiao Qing Dai, Robert C. Wright, Julie Hayward, Martijn van de Bunt, Tatsuya Kin, A. M. James Shapiro, Mark I. McCarthy, Anna L. Gloyn, Mark D. Ungrin, Jonathan R. Lakey, Norm M. Kneteman, Garth L. Warnock, Gregory S. Korbutt, Raymond V. Rajotte, Patrick E. MacDonald

**Affiliations:** Alberta Diabetes Institute, University of Alberta, LKS Centre, Edmonton, AB Canada T6G 2R3; Department of Pharmacology, University of Alberta, Edmonton, Canada; Department of Surgery, University of Alberta, Edmonton, Canada; Oxford Centre for Diabetes, Endocrinology, and Metabolism, University of Oxford, Oxford, UK; Wellcome Trust Centre for Human Genetics, University of Oxford, Oxford, UK; Oxford NIHR Biomedical Research Centre, Churchill Hospital, Oxford, UK; Faculty of Veterinary Medicine, University of Calgary, Calgary, Canada; Departments of Surgery and Biomedical Engineering, University of California, Irvine, USA; Department of Surgery, University of British Columbia, Vancouver, Canada; Department of Surgery, Surgical Medical Research Institute, HMRC, University of Alberta, Edmonton, AB Canada T6G 2S2

**Keywords:** Cryopreservation, Exocytosis, Human, Insulin, Ion channels, Islets, Secretion, Transplantation

## Abstract

**Aims/hypothesis:**

There are potential advantages to the low-temperature (−196°C) banking of isolated islets, including the maintenance of viable islets for future research. We therefore assessed the in vitro and in vivo function of islets cryopreserved for nearly 20 years.

**Methods:**

Human islets were cryopreserved from 1991 to 2001 and thawed between 2012 and 2014. These were characterised by immunostaining, patch-clamp electrophysiology, insulin secretion, transcriptome analysis and transplantation into a streptozotocin (STZ)-induced mouse model of diabetes.

**Results:**

The cryopreservation time was 17.6 ± 0.4 years (*n* = 43). The thawed islets stained positive with dithizone, contained insulin-positive and glucagon-positive cells, and displayed levels of apoptosis and transcriptome profiles similar to those of freshly isolated islets, although their insulin content was lower. The cryopreserved beta cells possessed ion channels and exocytotic responses identical to those of freshly isolated beta cells. Cells from a subset of five donors demonstrated similar perifusion insulin secretion profiles pre- and post-cryopreservation. The transplantation of cryopreserved islets into the diabetic mice improved their glucose tolerance but did not completely normalise their blood glucose levels. Circulating human insulin and insulin-positive grafts were detectable at 10 weeks post-transplantation.

**Conclusions/interpretation:**

We have demonstrated the potential for long-term banking of human islets for research, which could enable the use of tissue from a large number of donors with future technologies to gain new insight into diabetes.

## Introduction

The ability to preserve human islets for an extended period of time has several benefits. These may include increasing the practicality of islet transplantation for type 1 diabetes by improving the prospects for pre-transplant testing, simultaneous multiple donor transplants and simplified logistics. However, human islets are increasingly being recognised as a valuable research resource [1], and concerns have been raised about the future of access to human research islets [2]. Biobanking is one way to address this and to facilitate the progress of research into the mechanisms underlying diabetes. However, the successful storage of functional islets has not been achieved beyond 2 years—far too short a time for successful long-term biobanking.

Culturing islets extends their viable lifespan to a certain degree, but long-term maintenance of the phenotype has proved problematic [3, 4]. Schmied et al have reported the maintenance of endocrine cells to be limited to 60 days, with hormone secretion lost by day 28 [5], although Fraga et al observed glucose-stimulated insulin secretion following 2 months of culture [6]. The cryopreservation of islets has been considered to be a potential tool since the first demonstrations of the reversal of hyperglycaemia by frozen-thawed islets in rats used as a model of streptozotocin (STZ)-induced diabetes [7, 8]. Cryopreservation was initially undertaken with rodent and canine islets, and the cryopreservation of human islets was first reported in 1980 [9]. Using a protocol we had developed by 1989 [10, 11], we obtained reasonable results using islets cryopreserved for an average of 44 days, in which the insulin secretory responses were 79% of the pre-freezing levels [12]. The transplantation of a mixture of fresh and cryopreserved islets into patients with type 1 diabetes receiving kidney transplants demonstrated long-term graft function, with one patient becoming independent of insulin for 2.5 years [13, 14].

In the past 10 years, new cryopreservation protocols have been developed in an attempt to improve islet viability after thawing. These include pre- and post-culture with astragalosides, Sertoli cells, ductal epithelia, antioxidants and P38 MAP kinase (MAPK) inhibition [15–19]. Numerous cryoprotectants, including silk protein, DMSO, hydroxyethyl starch, glycerol and synthetic glycoprotein, have been investigated [20–23]. More recently, there has been a report of an increased duration of cryopreservation (3 months) and an assessment of post-thawing status including gene expression profiling, viability, insulin secretion and in vivo engraftment [24]. In addition, Misler et al have investigated the electrophysiological and secretory responses of islets following cryopreservation for up to 2 years [25].

Although the cryopreservation of human islets would be beneficial in enabling their future use, a true long-term banking of functional islets has not been reported. The University of Alberta is exceptional in terms of its large biobank of cryopreserved human islets [26]. Despite their success in clinical trials [13, 27], these frozen islets have, however, been deemed to be unsuitable for transplantation using the Edmonton protocol [28] owing to the presence of FBS. As such, preparations for which the donors had given research consent were designated as research islets. Here we assess the function of human islets obtained from this facility following cryopreservation for up to 21 years and demonstrate the potential for this approach to provide a source of high-quality, viable research tissue.

## Methods

### Human islets

We thawed and examined cryopreserved islet preparations from 43 human donors. These had been cryopreserved using the method developed by Rajotte et al [11] with DMSO as a cryoprotectant and had been stored under liquid-phase N_2_ for 17.6 ± 0.4 years. The mean age of the donors was 40.9 ± 2.0 years, with 47% male vs 53% female donors. Freshly isolated islets were obtained from the Alberta Diabetes Institute IsletCore and the Clinical Islet Laboratory at the University of Alberta. The mean age of the donors of these was 60.5 ± 3.0 years, with 56% male vs 43% female donors. All the studies were approved by the Human Research Ethics Board at the University of Alberta.

### Human islet culture

Cryopreserved islet preparations were thawed by rapid (150°C/min) warming to 4°C followed by the removal of DMSO with a sucrose gradient and serial dilution of cryoprotectant [11]. The islets were subsequently cultured overnight in CMRL 1066 (Corning, Tewksbury, MA, USA) supplemented with 0.5% BSA (Equitech-Bio, Kerrville, TX, USA), 1% Insulin-Transferrin-Selenium (Corning), 100 U/mL penicillin/streptomycin (Life Technologies, Burlington, ON, Canada) and l-glutamine (Sigma-Aldrich, Oakville, ON, Canada). Both the cryopreserved and the freshly isolated islets were cultured for an additional 24 h in low-glucose (1 g/L) DMEM supplemented with 10% FBS and 100 U/mL penicillin/streptomycin.

### Transcriptome analysis

Thawed islets (*n* = 32 donors) or freshly isolated islets (*n* = 18 donors) were hand-picked and RNA was extracted using Trizol reagent (Life Technologies). RNA sequencing was performed on an Illumina HiSeq 2000 (Illumina, San Diego, CA, USA) with 100 bp paired-end sequencing. Libraries were prepared using the NEBNext Ultra Directional RNA Library Prep Kit for Illumina (New England BioLabs, Hitchin, UK) with custom 8 bp indexes [29]. The TruSeq PE Cluster Kit v3 (Illumina) was used for cluster generation and the TruSeq SBS Kit v3 (Illumina) for sequencing. Sequenced reads were mapped to the human genome (GRCh37) with Tophat v2.0.12 (www.ccb.jhu.edu/software/tophat/index.shtml) [30] using Gencode v18 (www.gencodegenes.org) as the transcriptome reference. Expression was quantified using Cuffquant and Cuffnorm (http://cole-trapnell-lab.github.io/cufflinks/) [30] on default settings.

### Immunohistochemistry and TUNEL staining

Islets and islet grafts were fixed in Z-fix (Anatech, Battle Creek, MI, USA), embedded in paraffin and sliced into 5 μm sections. Immunostaining for insulin (Santa Cruz Biotechnology, Dallas TX, USA; or DAKO Canada, Burlington, ON, Canada) and glucagon (EMD Millipore, Billerica, MA, USA) was performed as previously described [31]. Apoptosis was assessed by in situ TUNEL (Roche Diagnostics, Laval, QC, Canada) according to the manufacturer’s instructions. Slides were coverslipped with prolong gold antifade with DAPI, visualised with an Axioscope II with AxioCamMRC and analysed using Axiovision 4.6 (Carl Zeiss, Gottingen, Germany).

### Electrophysiology

The islets were dispersed by shaking in dissociation buffer (Life Technologies) and plated in 35 mm dishes. The standard whole-cell technique was used with the sine+DC lock-in function of an EPC10 amplifier and Patchmaster software (HEKA Electronik, Lambrecht/Pfalz, Germany). Experiments were performed at 32–35°C. To measure the voltage-dependent K^+^ (Kv) currents, an intracellular solution was used containing (in mmol/l): 140 KCl, 1 CaCl_2_, 1 MgCl_2_, 10 HEPES, 10 EGTA and 3 ATP-Mg (pH 7.3 with KOH). The bath solution contained (in mmol/l): 135 NaCl, 5.4 KCl, 1 CaCl_2_, 1.2 MgCl_2_, 10 HEPES and 5 glucose (pH 7.3 with NaOH). To measure the capacitance, a bath solution was used that contained (in mmol/l): 118 NaCl, 20 tetraethylammonium chloride, 5.6 KCl, 1.2 MgCl_2_·6H_2_O, 2.6 CaCl_2_, 1 or 10 glucose and 5 HEPES (pH 7.4 with NaOH). The pipette solution for capacitance measurement contained (in mmol/l): 125 Cs-glutamate, 10 CsCl, 10 NaCl, 1 MgCl_2_·6H_2_O, 0.05 EGTA, 5 HEPES, 0.1 cAMP and 3 MgATP (pH 7.15 with CsOH). The patch pipettes, pulled from borosilicate glass and coated with Sylgard (World Precision instruments, Sarasota, FL, USA), had resistances of 3–4 MΩ when filled with pipette solution. The whole-cell Kv current and capacitance responses were normalised to the initial cell size and expressed as pA/pF and fF/pF, respectively. The beta cells were positively identified after the experiment by insulin immunostaining.

### Insulin secretion

Static insulin secretion measurements were performed at 37°C in KRB (in mmol/l: 115 NaCl, 5 KCl, 24 NaHCO_3_, 2.5 CaCl_2_, 1 MgCl_2_, 10 HEPES and 0.1% BSA; pH 7.4). Twenty islets per group were pre-incubated for 2 h in 1 mmol/l glucose KRB. These were subsequently incubated for 1 h in KRB with 1 mmol/l glucose, followed by 1 h with 16.7 mmol/l glucose and then 1 h with 16.7 mmol/l glucose plus 20 mmol/l KCl. The islets were extracted with acid/ethanol to determine their insulin content. The samples were stored at −20°C and assayed for insulin using an electrochemiluminescence assay (Meso Scale Discovery, Rockville, MD, USA).

Insulin secretion was measured pre- and post-cryopreservation in five donors by perifusion assay. Pre-cryopreserved islets were perfused with Hanks’ Balanced Salt Solution (containing 2.8 or 28 mmol/l glucose), and samples were collected and assayed for insulin using radioimmunoassay. Post-thaw insulin perifusions were performed using a PERI-4.2 perifusion machine (Biorep Technologies, Miami, FL, USA). Twenty islets per chamber were perfused with KRB (containing 2.8 or 28 mmol/l glucose) at a flow rate of 250 μl/min with samples collected at 2 min intervals.

### Islet transplantation

Male B6.129S7-*Rag1*^tm1Mom^/J (B6/*Rag*^−/−)^ mice (Jackson Laboratory, Bar Harbor, ME, USA) were rendered diabetic by the intraperitoneal injection of streptozotocin (STZ; 185 mg/kg). Three days after the induction of diabetes, islets thawed after cryopreservation (2,000 or 4,000 islet equivalents [IEQ]) or freshly isolated islets (3,000 IEQ) were transplanted under the kidney capsules of the mice [32]. Their body weight and blood glucose levels were monitored over 10 weeks. Blood glucose and plasma insulin (Meso Scale Discovery) were both measured in response to an oral glucose load (3 mg/g body weight) pre- and post-nephrectomy, which occurred at week 10. The islet graft was extracted in acid-ethanol to determine the insulin content or embedded and sectioned for immunohistochemistry. All murine experiments were approved by the Animal Care and Use Committee at the University of Alberta.

### Data analysis

Data were analysed using FitMaster (HEKA Electronik) and Origin 9.1 (OriginLab, Northampton, MA, USA) and compared using multiple ANOVA and the Student’s *t* test. Data are expressed as mean ± SE, and *p* < 0.05 was considered significant.

## Results

### Human islets following long-term cryopreservation and thawing

**Fig. 1:**
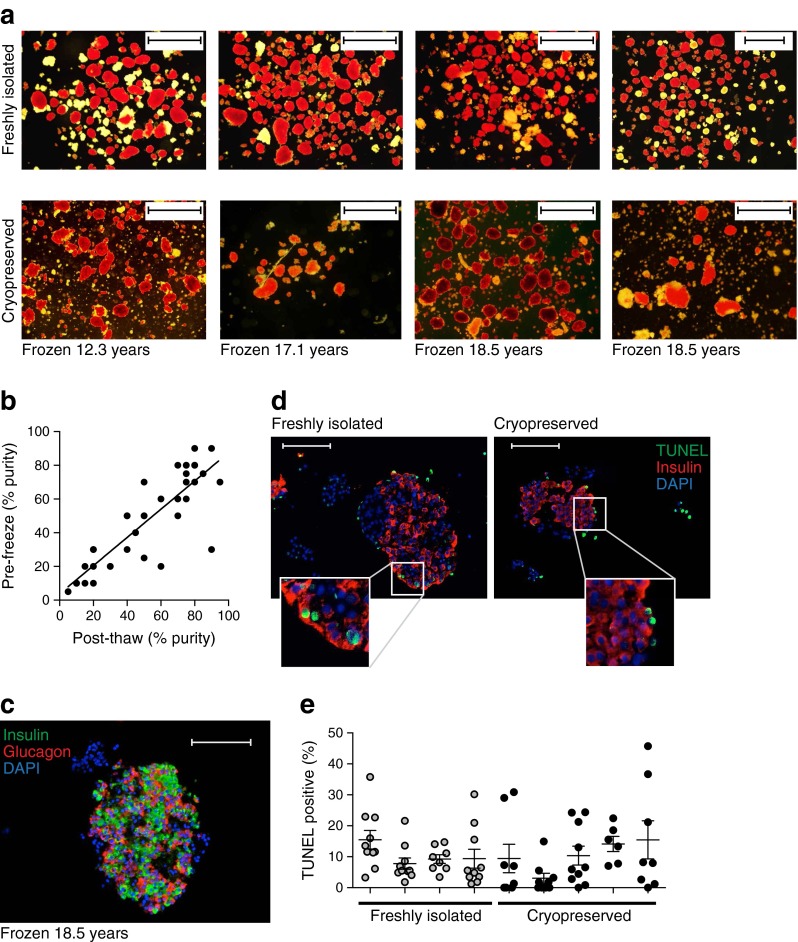
Human islets following long-term cryopreservation. (**a**) Islets were stained with dithizone immediately following isolation or on thawing following long-term cryopreservation. Scale bar, 1 mm. (**b**) Correlation of islet purity estimates at isolation (Pre-freeze) and after thawing (Post-thaw). (**c**) Representative image of a long-term cryopreserved islet, immunostained for insulin (green) and glucagon (red), with nuclei stained with DAPI (blue). Scale bar, 100 μm. (**d**) Representative image of a freshly isolated and a cryopreserved islet stained for insulin (red), TUNEL (green) and DAPI (blue). Scale bar, 100 μm. (**e**) Percentage of apoptotic (TUNEL-positive) cells within freshly isolated islets from four donors (grey circles) and cryopreserved islets from five donors after thawing (black circles). Each circle represents a separate image set (17,185 cells)

Human islet preparations (*n* = 43, frozen for 17.6 ± 0.4 years) were rapidly thawed using the method described by Rajotte et al [11] to limit osmotic damage. The cells were immediately stained with dithizone (Fig. 1a). Estimates of islet purity pre- and post-cryopreservation were positively correlated (slope = 0.84, *r*^2^ = 0.73, *p* < 0.001; Fig. 1b), and cryopreservation did not significantly decrease the purity (51.2 ± 4.4 vs 47.2 ± 4.1%, *n* = 41). Immunofluorescence staining revealed the expression of both insulin-positive beta cells and glucagon-positive alpha cells (Fig. 1c), which appeared to be similar in their distribution to previous reports [33]. Apoptosis, assessed by TUNEL, did not differ between freshly isolated (9.4 ± 1.2%, *n* = 4 donors) and cryopreserved islets (8.9 ± 1.8%, *n* = 5 donors; Fig. 1d, e) and was similar to values previously reported [34–37].

### Cryopreserved beta cells retain ion channel function and exocytotic responsiveness

**Fig. 2:**
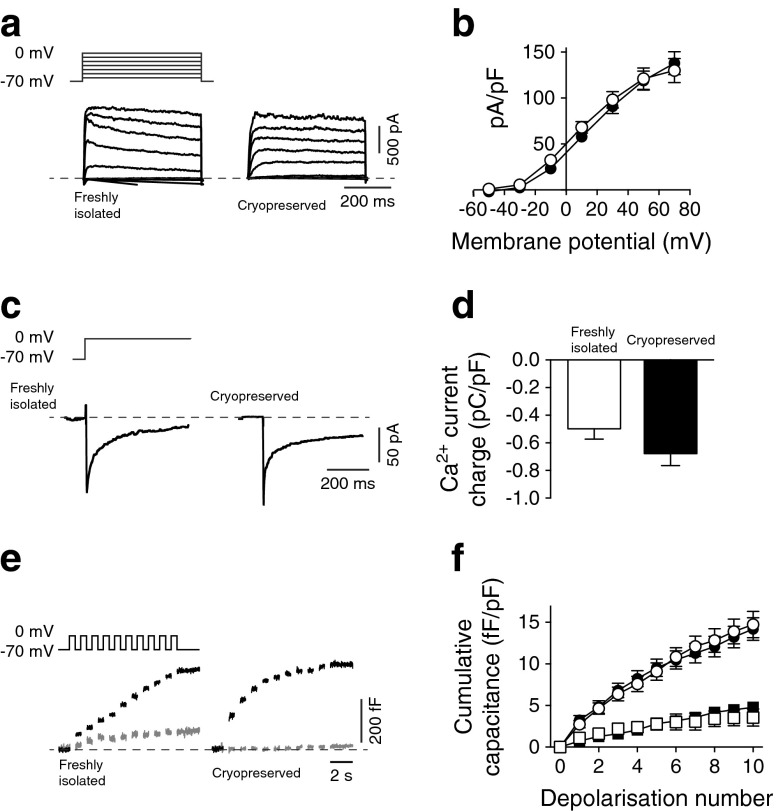
Single cell function is maintained in long-term cryopreserved human beta cells. (**a**, **b**) Representative Kv currents and current–voltage relationships from freshly isolated (white circles) and long-term cryopreserved (black circles) human beta cells. (**c**, **d**) Representative VDCCs and Ca^2+^-charge entry during depolarisation in freshly isolated (white bar) and long-term cryopreserved (black bar) human beta cells. (**e**, **f**) Representative exocytotic responses, shown as increases in membrane capacitance (i.e. surface area) in response to a series of membrane depolarisations, and summarised data from freshly isolated (white symbols) and long-term cryopreserved (black symbols) human beta cells in response to 1 mmol/l (squares) and 10 mmol/l (circles) glucose. In all experiments, the beta cells were positively identified by immunostaining for insulin

A critical component of beta cell function is the electrical activity mediated by the ion channels, leading to Ca^2+^ influx and the exocytosis of insulin-containing granules [38, 39]. Whole-cell patch-clamp was used to assess the Kv current, a key regulator of action potentials and insulin secretion [40, 41]. The Kv currents from cryopreserved (*n* = 15 donors, 45 cells) and freshly isolated (*n* = 3 donors, 15 cells) beta cells identified by positive insulin immunostaining were similar (138.6 ± 11.7 vs 130.3 ± 11.3 pA/pF; Fig. 2a, b).

Voltage-dependent Ca^2+^ channels (VDCCs) underlie the influx of Ca^2+^ on glucose-stimulated membrane depolarisation to trigger the release of insulin granules [41]. VDCC currents exhibited similar activity in cryopreserved beta cells (−0.68 ± 0.09 pC/pF, *n* = 8 donors, 31 cells) and freshly isolated beta cells (−0.50 ± 0.07 pC/pF, *n* = 6 donors, 24 cells; Fig. 2c, d). Although the beta cells from the cryopreserved islets tended to show a larger Ca^2+^ entry in response to depolarisation, this was not statistically significant.

The fusion of insulin granules with the cell membrane can be measured as an increase in cell capacitance in response to a train of depolarisations [42]. The magnitude of this response is sensitive to glucose level in healthy beta cells [43], probably reflecting a metabolism-driven increase in insulin granule trafficking or priming. The cryopreserved beta cells had intact exocytotic responses and retained their sensitivity to glucose (5.0 ± 0.6 fF/pF at 1 mmol/l glucose vs 15.0 ± 1.4 fF/pF at 10 mmol/l glucose, *n* = 9 donors, 46–70 cells), which was similar to that seen in healthy, freshly isolated beta cells (4.1 ± 0.9 fF/pF at 1 mmol/l glucose vs 17.0 ± 2.2 fF/pF at 10 mmol/l glucose, *n* = 6 donors, 20–26 cells; Fig. 2e, f).

### Cryopreserved human islets exhibit glucose-stimulated insulin secretion

**Fig. 3:**
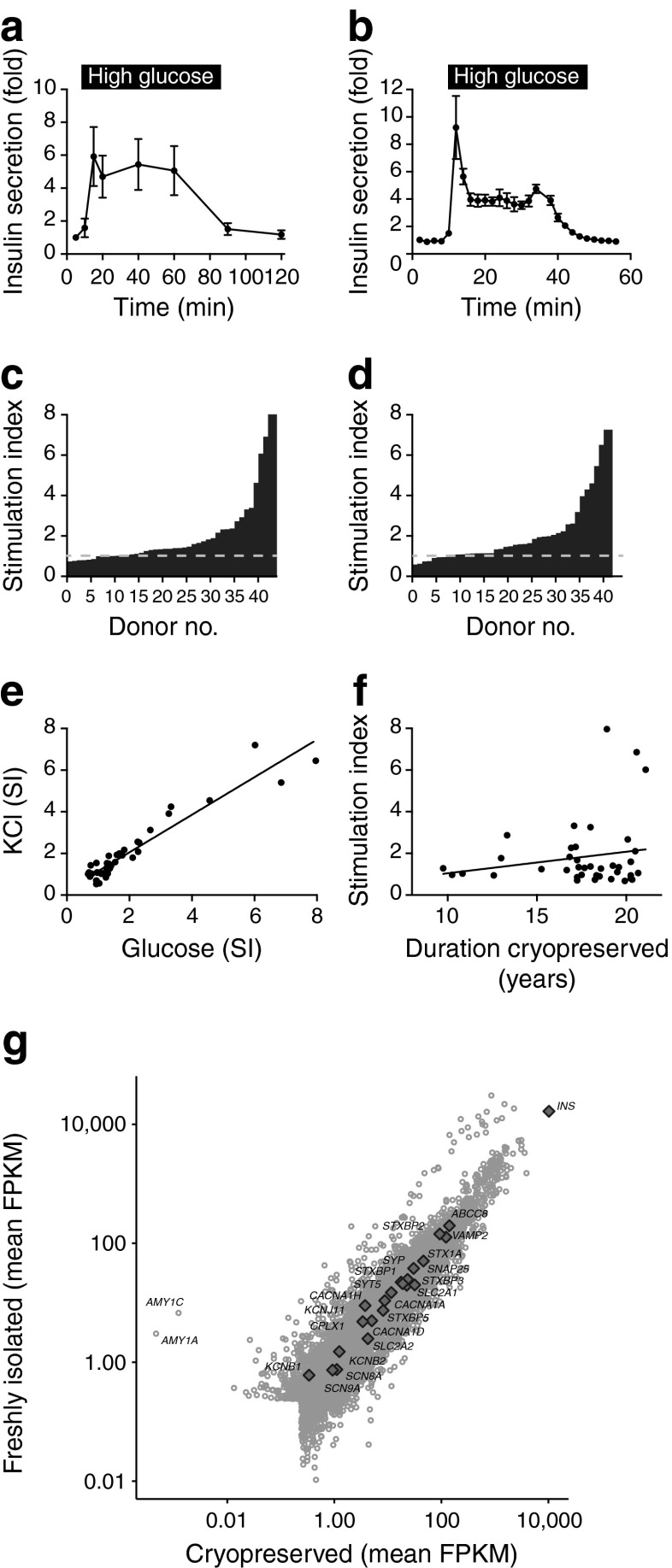
Function of intact cryopreserved human islets in vitro. (**a**, **b**) Insulin secretion measured by perifusion in response to 28 mmol/l glucose (high glucose) from islets following isolation (**a**) and then from the same preparations after thawing approximately 20 years later (**b**). (**c**, **d**) Static insulin secretory responses of long-term cryopreserved islets to 16.7 mmol/l glucose alone (**c**) or with 20 mmol/l KCl (**d**). (**e**, **f**) The secretory responses to KCl and glucose were positively correlated (**e**), but secretory competence did not correlate with the duration of cryopreservation (**f**). (**g**) Transcriptome analysis demonstrated a high degree of correlation of transcript expression between cryopreserved and freshly isolated islets (Spearman’s *ρ* = 0.94). The expression level of several beta cell function genes is indicated. FPKM, fragments per kilobase of exon per million fragments mapped; SI, stimulation index

Our electrophysiological studies indicated that the ion channels and exocytotic machinery necessary to elicit insulin granule fusion remained intact following cryopreservation. We were able to directly compare the secretory profile of the islets from five donors before cryopreservation (Fig. 3a) and then after thawing almost 20 years later (Fig. 3b). Glucose-stimulated (28 mmol/l) secretion was remarkably similar in magnitude both pre- and post-cryopreservation, with a modest difference in the profile that was likely to be attributable to improved methodology or increased sampling frequency.

The secretion of insulin from the islets was measured following static incubation with 1 mmol/l glucose, 16.7 mmol/l glucose or 16.7 mmol/l glucose plus 20 mmol/l KCl. Glucose and KCl stimulated insulin secretion with stimulation indices of 1.90 ± 0.24 and 1.96 ± 0.24, respectively (*n* = 41–43 donors). The stimulation of insulin secretion in response to glucose (*n* = 43) and KCl (*n* = 41) reached statistical significance (at *p* < 0.05) for 37.2% and 41.5% of donors, respectively (Fig. 3c, d). The total insulin content of the cryopreserved islets (11.1 ± 1.4 ng/islet, *n* = 43) was significantly lower than what we can observe in freshly isolated islets today (24.3 ± 2.3 ng/islet, *n* = 56, *p* < 0.001). However, due to a lack of historical data on the insulin content, we do not know whether this was reduced at the time of isolation or by the cryopreservation process per se.

Although there was a significant linear correlation (*r*^2^ = 0.91, *p* < 0.001) between glucose sensitivity and KCl sensitivity (Fig. 3e), the duration of cryopreservation did not correlate (*r*^2^ = 0.03) with the glucose-stimulated insulin secretion (Fig. 3f). This preserved functional capacity is consistent with our assessment of transcript expression profiles by RNA sequencing (Fig. 3g) and our demonstration of a similar expression of the exocytotic and ion channel genes. There is strong correlation (Spearman’s *ρ* = 0.94) between the transcriptomes of the cryopreserved and the fresh islets. The two outliers observed in Fig. 3g are *AMY1A* and *AMY1C*, suggesting differences in islet purity (although this is likely to be due to the hand-picking of cryopreserved islets prior to the transcriptome analysis).

### Partial functioning of cryopreserved islets transplanted into diabetic mice

**Fig. 4:**
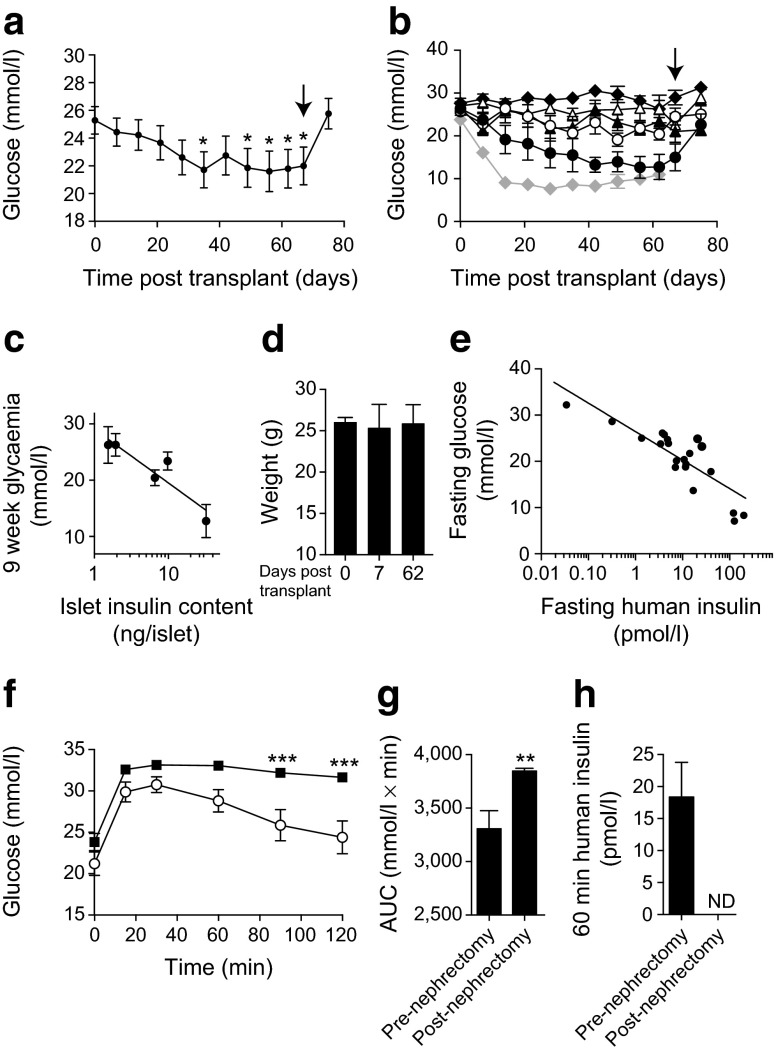
Long-term cryopreserved human islets are partially functional in vivo. (**a**) Sub-capsular grafts of cryopreserved human islets (2,000–4,000 IEQ) from five separate donors produced a marginal improvement in blood glucose level in STZ-diabetic B6/*Rag* ^−/−^ mice, which was reversed by removal of the graft-bearing kidney (arrow). (**b**) Glycaemic responses to grafts from each of five donors are shown, compared with the effect of freshly isolated human islets (3,000 IEQ; grey line). The grafts were removed at the time indicated by the arrow. (**c**) The success of reversal of diabetes at 9 weeks correlates with the insulin content of the cryopreserved islet preparations. (**d**) Body weight was maintained in the STZ-diabetic B6/*Rag* ^−/−^ mice following the transplantation of cryopreserved human islets. (**e**) Human insulin was detectable in fasted STZ-diabetic B6/*Rag* ^−/−^ recipient mice, and levels correlated negatively with fasting blood glucose levels. (**f**–**h**) Long-term cryopreserved islets improved intraperitoneal glucose tolerance (white circles) and the AUC of the glucose response in STZ-diabetic B6/*Rag* ^−/−^ mice, as well as producing human insulin (**h**). After removal of the engrafted kidney (black squares), the glucose excursion was elevated and circulating human insulin was not detectable (ND). *p < 0.5, **p < 0.01 and ***p < 0.001 compared with time = 0 or the pre-nephrectomy control

To ascertain the extent to which long-term cryopreserved islets can function in an in vivo model, we transplanted 2,000–4,000 IEQ from five donors (cryopreserved for 16.6 ± 1.1 years, stimulation index 1.9 ± 0.4, four to five recipient animals each) under the kidney capsule of STZ-induced diabetic B6/*Rag*^−/−^ mice. Transplantation significantly decreased blood glucose levels (*n* = 5 donors, 23 animals, *p* < 0.05) but did not restore normoglycaemia (Fig. 4a). The success of engraftment varied between donors, with only one group achieving glucose levels similar to those of freshly isolated islets (*n* = 5 donors, 11 mice), although this occurred after a prolonged delay (Fig. 4b). The success of the engraftment did not correlate with the stimulation index but did correlate significantly (*r*^2^ = 0.84, *p* < 0.05) with the insulin content of the donor islets (Fig. 4c). In all cases, however, the transplanted cryopreserved islets were sufficient to maintain the animal’s body weight (Fig. 4d). Human insulin was detected in most recipient mice following fasting and was significantly (*r*^2^ = 0.73, *p* < 0.001) correlated with improvements in fasting glucose levels (Fig. 4e).

In mice receiving grafts of cryopreserved islets, OGTTs were performed at week 10 and again 1 week following graft removal. Although the animals were hyperglycaemic, their blood glucose excursions were significantly improved by the graft (*n* = 23, *p* < 0.001; Fig. 4f), and graft removal increased the area under the glucose curve (*n* = 23, *p* < 0.01) (Fig. 4g). Since the maximum value of the capillary glucose meter is 33.3 mmol/l, the true blood glucose values post-nephrectomy are likely to be higher than reported. Circulating human insulin at 60 min following glucose challenge, which was detectable in recipient mice pre-nephrectomy, was absent following graft removal (Fig. 4h).

**Fig. 5:**
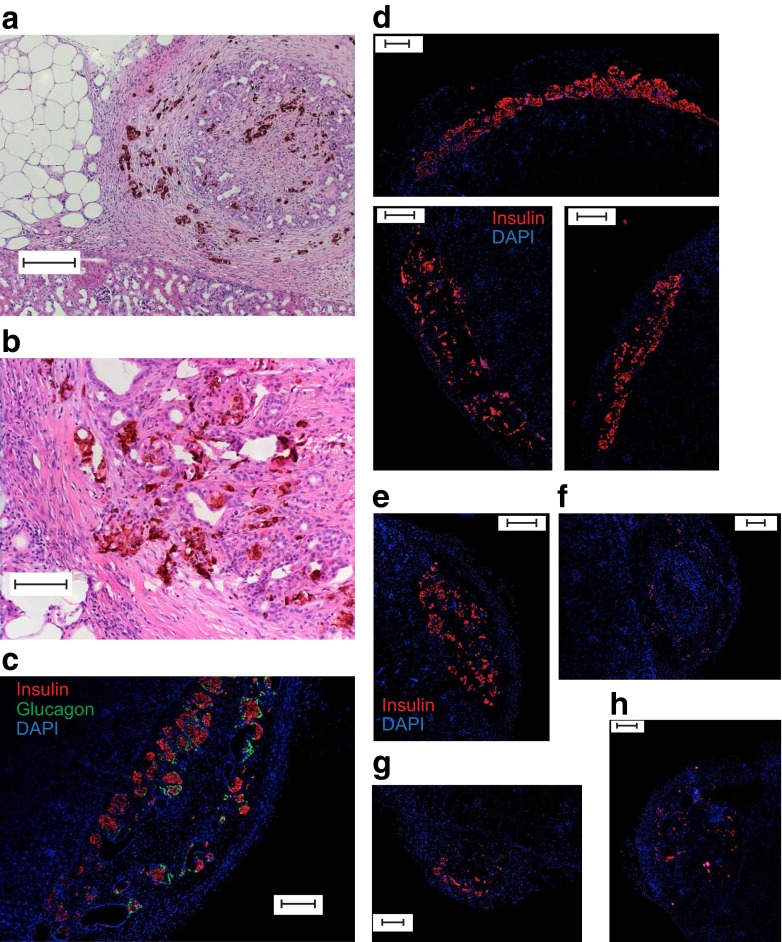
Human insulin is detectable in grafts of long-term cryopreserved human islets after 10 weeks. (**a**, **b**) Anti-insulin HRP/DAB staining of renal sub-capsular grafts of long-term cryopreserved human islets. (**c**) Insulin (red) and glucagon (green) immunofluorescence and DAPI staining (blue) in a section of a graft of cryopreserved islets removed after 10 weeks. (**d**–**h**) Insulin immunostaining (red) and DAPI staining (blue) of grafts of freshly isolated islets from three separate donors (**d**) and long-term cryopreserved islets from four separate donors (**e**–**h**). The cryopreserved islets shown were from donors that reduced the blood glucose level in recipient mice to 12.4 (**e**), 20.4 (**f**), 26.3 (**g**) and 23.4 (**h**) mmol/l. Scale bar, 100 μm (**a**) or 200 μm (**b**–**e**)

Anti-insulin-horseradish peroxidase (HRP)/3,3′-diaminobenzidine (DAB) (Fig. 5a, b) and immunofluorescence (Fig. 5c) revealed the retention of human insulin in cryopreserved islet grafts from four out of five donors after 10 weeks. However, although grafts of freshly isolated islets showed strong insulin-positive staining (Fig. 5d), the insulin staining of cryopreserved islet grafts appeared to be weaker (Fig. 5e–h), consistent with the lower islet insulin content observed above. The total insulin content of the grafts of cryopreserved islets was 399.1 ± 119.4 ng/graft (*n* = 12 grafts, five donors). The level of apoptosis did not differ between the fresh (4.3 ± 1.6%, *n* = 10) and the cryopreserved (5.4 ± 2.0%, *n* = 6) grafts.

## Discussion

This is the first report of human islet function following extended cryopreservation in a large cohort of donors and suggests that the potential storage duration of functional islets can be increased by an order of magnitude. We have assessed various steps in the stimulus–secretion coupling pathway and provide data indicating that long-term cryopreservation provides a source of human islet material for research that retains many physiologically relevant properties. Access to human research islets remains a limiting factor in many scientific studies [2], and the inherent difficulties of distribution and culture time only add further to the value of any human islet tissue obtained. Biobanking has the potential to overcome many of these issues, and the successful retention of physiological function in frozen-thawed islets is desirable.

When assessing beta cell function, several critical components of stimulus–secretion coupling must be considered. These include ion channel activity, which underlies the electrical signal produced in response to glucose stimulation, Ca^2+^ influx and the exocytotic machinery responsible for the release of insulin granules [38, 39]. Ultimately, glucose-stimulated insulin secretion and the ability to restore physiological glucose homeostasis are of key significance.

Our patch-clamp studies revealed that key ion conductances underlying beta cell excitability remain identical to those observed in freshly isolated beta cells following up to 21 years of cryopreservation. In addition, measurement of the exocytotic capacity and glucose sensitivity of single beta cells demonstrates responses that are indistinguishable from those of freshly isolated beta cells. These findings expand on those of Misler et al, who previously measured electrical activity and exocytosis in human islets cryopreserved for a much shorter period in a small cohort of donors [25]. Our data indicate that cryopreserved human islets can serve as an appropriate physiological model in at least some cellular mechanistic studies.

While these underlying cellular processes remain intact, in vitro insulin secretion and islet insulin content appear to be more variable in the cryopreserved islets. The mean glucose stimulation index of 1.90 ± 0.24 (*n* = 43) is low compared with the values we typically observe today with freshly isolated human islets (9.53 ± 0.92, *n* = 56; range 1.04–33.83). A reduction in insulin secretory capacity has frequently been reported following the cryopreservation of human islets [16, 22, 25, 44, 45]. However, given the limited historical data available to us, we could only make a direct pre- and post-cryopreservation comparison for five donors (Fig. 3a, b). As the secretory profiles for these donors were similar before and after freezing, it is difficult to conclude that cryopreservation per se caused a reduced secretory response in our study. Notably, the return to baseline secretion in the perifusion, which is difficult to achieve in rat islets cryopreserved using other protocols [10], suggests that these islets were viable. Finally, the transcriptome profiles of fresh and cryopreserved islets were similar, including the expression of genes that correlate with the measurements made here: for insulin (*INS*), Kv currents (i.e. *KCNB1* and *KCNB2*), Ca^2+^ currents (i.e. *CACNA1A*, *CACNA1H* and *CACNA1D*) and exocytosis (i.e. *VAMP2*, *STX1A*, *SNAP25*, *CPLX1* and *SYP*).

Our transplantation of cryopreserved islets into STZ-induced diabetic mice revealed that in vivo function varies and correlates with islet insulin content. As above, however, because we do not have data on the pre-freezing insulin content, we cannot say whether cryopreservation itself reduces the insulin content or whether this was already lower prior to freezing. Overall, the cryopreserved islets performed relatively poorly in their ability to normalise blood glucose level in vivo. The maintenance of the recipients’ body weights and the presence of circulating human insulin, which correlated inversely with blood glucose level, indicate, however, that the cryopreserved islets maintained some function in vivo. This is supported by the OGTTs, which showed that the grafts were limiting the blood glucose excursion. Most grafts retained endocrine cell expression for the full 10 weeks. Indeed, graft-removing nephrectomy completely abolished the plasma insulin and resulted in severe glucose intolerance. Thus, we conclude that the cryopreserved human islet grafts retained at least some function in vivo, but the reversal of diabetes was limited by the reduced insulin content of these islets.

In summary, we have demonstrated that long-term cryopreserved islets may be suitable for cellular studies of the mechanisms underlying stimulus–secretion coupling in beta cells, although further work is required to determine the potential effects of long-term cryopreservation on insulin content that may limit their in vivo efficacy. The progress of science and technology is rapid. These human islets were isolated and cryopreserved in an era when many of today’s modern techniques were undeveloped or prohibitive in terms of cost. In less than two decades, the research potential of this tissue has dramatically increased. The ongoing banking of human islets is therefore important in order to preserve tissue for the evaluation of currently unidentified targets and pathways using advanced technologies, which will facilitate future research.

## Abbreviations

DAB: 3,3′-Diaminobenzidine
HRP: Horseradish peroxidase
IEQ: Islet equivalents
Kv: Voltage-dependent K^+^
VDCC: Voltage-dependent Ca^2+^ channel
